# Restoration of CpG Methylation in The *Egf* Promoter Region during Rat Liver Regeneration

**DOI:** 10.22074/cellj.2015.20

**Published:** 2015-10-07

**Authors:** Li Deming, Li Ziwei, Guo Xueqiang, Xu Cunshuan

**Affiliations:** 1 Key Laboratory for Cell Differentiation Regulation, Xinxiang, China; 2 College of Life Science, Henan Normal University, Xinxiang, China

**Keywords:** Epidermal Growth Factor, Methylation, Liver Regeneration

## Abstract

Epidermal growth factor (EGF) is an important factor for healing after tissue damage in diverse experimental models. It plays an important role in liver regeneration (LR). The objective of this experiment is to investigate the methylation variation of 10 CpG sites in the *Egf* promoter region and their relevance to *Egf* expression during rat liver regenera- tion. As a follow up of our previous study, rat liver tissue was collected after rat 2/3 partial hepatectomy (PH) during the re-organization phase (from days 14 to days 28). Liver DNA was extracted and modified by sodium bisulfate. The methylation status of 10 CpG sites in *Egf* promoter region was determined using bisulfite sequencing polymerase chain reaction (PCR), as BSP method. The results showed that 3 (sites 3, 4 and 9) out of 10 CpG sites have strikingly methylation changes during the re-organization phase compared to the regeneration phase (from 2 hours to 168 hours, P=0.002, 0.048 and 0.018, respectively). Our results showed that methylation modification of CpGs in the *Egf* promoter region could be restored to the status before PH operation and changes of methylation didn’t affect *Egf* mRNA expression during the re-organization phase.

## Introduction

It is well known that liver has an extraordinary capacity to regenerate itself after surgical resections, toxic injury or infections (1). Two-thirds partial hepatectomy (PH) in rodents provides an *in vivo* experimental model for studying liver regeneration (LR) (2,3). Hypertrophy of hepatocytes occurs in a few hours after PH followed by cell proliferation. Thus “compensatory hyperplasia” may describe this phenomenon more accurately (4). Even though nearly all remaining hepatocytes enter into S phase, only about half of them would divide and the lost liver mass can be restored within 5-7 days (5,6), which is referred as regeneration phase. Then a slow tissue remolding process takes place for several weeks to reorganize the newly regenerated tissue into the typical liver histology (7), which is referred as re-organization phase. 

It has been demonstrated that numerous genes and signals pathways are activated after PH to regulate the hypertrophy and proliferation of hepatocytes in a synchronous manner (1,6,8). Epidermal growth factor (EGF) has been reported to play an important role in rat LR. EGF is a polypeptide composed of 53 amino acids (9). It can be secreted into the lumen of the duodenum by Brunner’s glands and reaches liver via portal circulation after PH (3,10). Binding of EGF to its high affinity receptor (EGFR) induces the receptor to undergo homoor hetero-dimerization, which activates tyrosine kinase activity and stimulates multiple pathways of signal transduction including the RAS/RAF/ MEK/ERK1/2, the phospholipase-C (PLC)-gamma/protein kinase C (PKC), the phosphoinositide3-kinase (PI3K)/Akt, signal transducers and activators of transcription (STAT), and the nuclear factor kappa B (NF-kB) cascades. As results, the expression of array genes can be changed, which in turn affects a variety of physiological processes, such as cell growth, proliferation, regeneration, differentiation, and wound repair (11,14). 

Methylation is a covalent inherited modification of mammalian genomic DNA and occurs predominantly in the context of CpG dinucleotides (15,16). In general, CpG methylation in a promoter or enhancer region has a correlation with gene expression, which may directly inhibit the binding of certain transcriptional regulators to their cognate DNA sequences or indirectly by favoring the formation of repressive chromatin by methyl-CpG binding proteins, whereas methylation within gene body is positively correlated with gene expression (17,20). In higher eukaryotes, DNA methylation is critical for a variety of cellular activities such as genome stability and defense, genomic imprinting, X chromosome and transposon inactivation, paramutation, carcinogenesis and aging (21,23). Changes of methylation in acute response have been reported in recent years (24,27). We previously investigated methylation modification during the first week (from 2 hours to 168 hours) of rat LR and found that methylation change of 4 CpG sites (28). In order to better understand the role of methylation in the *Egf* promoter region in the regulation of rat LR, the current study is designed to follow-up methylation changes during the re-organization phase of rat LR. The results presented below reveal that the methylation at these sites can be restored to the status before PH operation during re-organization phase. 

## Animals treatment and DNA isolation

Sprague-Dawley rats are maintained at the Animal Center of Henan Normal University, Henan, China. Rats are raised in standard laboratory conditions (temperature 22 ± 2˚C, relative humidity 50-60%, and illumination 12 hours/day), with free access to standard rodent chow and distilled water. For this study, 12 healthy rats, 8-week old and weight of 230 ± 20 g, were randomly divided into 6 groups, 2 rats per group. Three groups were designated for 2/3 PH, three groups for sham-operation (SO). PH was performed according to Higgins and Anderson (2). Briefly, rats were anesthetized by pentobarbital sodium and sacrificed after PH, at days 14, 21 and 28, respectively. The regenerating liver was collected and stored at −80˚C before ready to use. The SO group underwent the same operation procedure, i.e. abdominal cavity was opened and liver lobes were flipped, but no liver lobes were excised. Genomic DNA was prepared from the liver tissue by proteinase K digestion and phenol/chloroform extraction following the method of Sambrook and Russell (29). All animal experimental procedures were conducted according to the Animal Protection Law of China and conformed to animal Ethics. 

## Primer design and bisulfite sequencing polymerase chain reaction (BSP)

**Fig.1:**
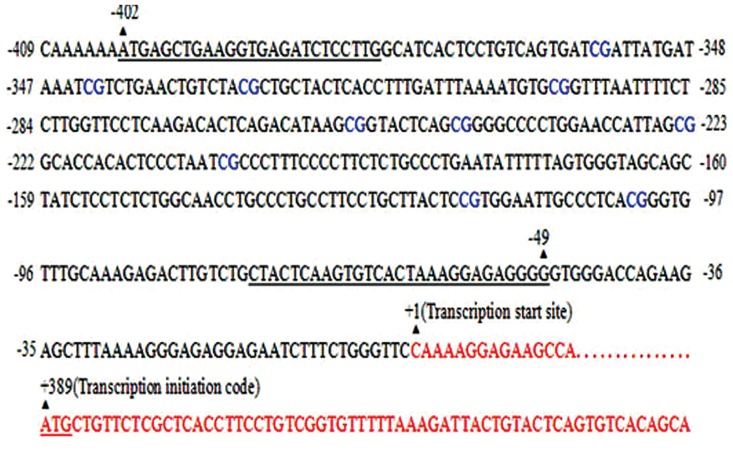
Schematic view of primer positions in the *Egf* promoter region. Primers designed for Bisulfite Sequencing PCR (BSP) by MethPrimer software and 10 CpG sites close to transcription start site are located in the -49 bp to -402 bp of the *Egf* promoter region. A 354 bp fragment was amplified by the BSP primers. *Egf*; Epidermal growth factor and PCR; Polymerase chain reaction.

The 1000 bp sequence of rat *Egf* promoter region was input to MethPrimer software (30) for bisulfite sequencing primer design. The primers used were 5΄-ATGAGTTGAAGGTGAGATTTTTTTG-3΄ (sense), and 5΄-CCCCTCTCCTTTAATAACACTTAAATAA-3΄ (antisense), which covers 354 bp from -49 to -402 from the transcription start site, with 10 of CpG sites in the *Egf* promoter region (Fig .1). DNA (500 ng) 

was modified with sodium bisulfite using EpiTect Bisulfite kit (Qiagen, Germany). Polymerase chain reaction (PCR) was performed for 40 cycles: 95˚C for 30 seconds, 56˚C for 30 seconds, and 72˚C for 30 seconds. Amplified bisulfite-sequencing PCR products were purified using the PCR purification kit (Dingguo Company, China) and inserted into pMD18-T vector (Takara Co., Dalian, China). The vector was then transformed into competent JM109 E. coli cells and 10 of positive clones were sequenced in each sample. 

### Reverse transcription and real-time quantitative polymerase chain reaction

Total RNA was extracted using Trigol (Dingguo Company, China) according to the supplier’s manual. cDNAs were generated using random primers and a reverse transcription kit (Promega Co, China). Real time quantitative polymerase chain reaction (RTQ-PCR) was performed using *Egf* specific primers based on the published *Egf* sequence (NM_012842.1): 5΄-ACCAACACGGAGGGAGGCTACAA-3΄ (forward) and 5΄-GCGGTCCACGGATTCAACATACA-3΄ (reverse). *Gapdh* (NM_017008.4) was included as control with a forward primary sequence of 5΄-CACGGCAAGTTCAACGGCACAGTCA-3΄ and reverse primary sequence of 5΄-GTGAAGACGCCAGTAGACTCCACGAC-3΄. Real-time quantitative PCR was performed with SYBR_Green I (Invitrogen, USA) using Rotor-Gene 3000 (Corbett Robotics, Australia). Thermal cycling was carried out at 95˚C for 2 minutes, followed by 40 cycles at 95˚C for 30 seconds, 59˚C for 30 seconds, and 72˚C for 30 seconds. Results were quantified using the software of the Rotor-Gene 3000. Each sample was performed in triplicate. The level of *Egf* expression was measured using the 2 ^-ΔΔCt^method (31). 

### Statistical analysis

Sequence alignment was performed by means of the software BiQ Analyzer (32). Statistical analysis was conducted using SPSS 13.0 software (SPSS Inc., Chicago, USA). The independent-samples t test was used to compare the difference between the PH and control groups. It was considered statistically significant if P value<0.05. 

Sequence alignment was performed by means of the software BiQ Analyzer (32). Statistical analysis was conducted using SPSS 13.0 software (SPSS Inc., Chicago, USA). The independent-samples t test was used to compare the difference between the PH and control groups. It was considered statistically signiﬁcant if P value<0.05.

Based on the software alignment, the percentage of methylation at total 10 CpG sites within the *Egf* promoter region was obtained from PH and SO groups at the indicated time points (Table 1). The methylation level at CpG1 and CpG2 was very low or undetectable. Methylation percentage of other sites ranged from 20 to 100%, with majority sites at high methylation level. For the PH group only, the methylation levels at CpG3, CpG4, and CpG9 sites were strikingly different in the re-organization phase (days 14, 21 and 28) compared to the regeneration phase (from 2 hours to 168 hours or days 7 with P=0.002, 0.048 and 0.018, respectively). The methylation percentage of CpG3 was increased, whereas the methylation percentage of CpG4 and CpG9 was decreased. No signiﬁcant methylation difference in the SO group was observed between the re-organization phase and the regeneration phase. The methylation levels at the selected 10 CpG sites were found no signiﬁcant differences between PH and SO groups during the re-organization phase (days 14, 21 and 28), yet 4 sites (CpG3, CpG4, CpG7 and CpG8) were found with striking differences between the two groups during the regeneration phase (from 2 hours to 168 hours or days 7) (28). This showed methylation modification of CpGs in the *Egf* promoter region could be restored to the SO status during the reorganization phase. The methylation percentage at site 3 only had striking difference in re-organization phase compared to the normal phase or 0 hour (0 hour total methylation percentage is 80.8%; online Supplementary Material) (28) in both the PH group and the SO group (P<0.05), the methylation percentage for all CpG sites was restored to a similar level as that before operation during the reorganization phase in these two groups, whereas the methylation percentage of CpG3 was not restored completely. Perhaps it needs longer time to restore, or it indicates subtle difference between regeneration tissue and normal tissue. The current finding suggests that methylation of CpGs in the *Egf* promoter region can be restored during the re-organization phase and changes of methylation may affect the progression of LR and re-organization.

**Fig.2:**
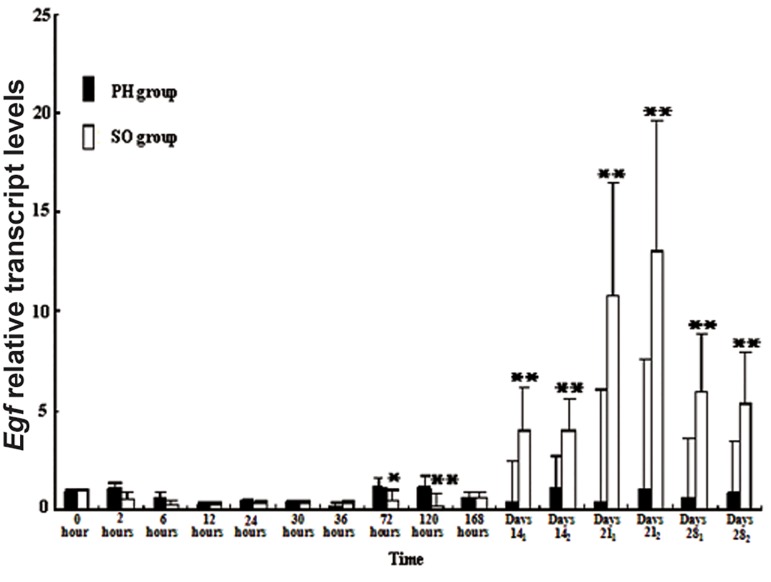
Changes of *Egf* mRNA level during the time course of rat LR. Rat liver tissue was collected after PH and SO at the indicated time point for RNA extraction. *Egf* mRNA level was detected by RTQ-PCR and the results were normalized against the internal *Gapdh* control. The previously published data (0-168 hours) were cited as the mean ± SD of the samples with triplicate RTQ-PCR experiments (hours). For this study, each data entry (days 14_1_ to days 28_2_) was obtained from one rat, for example, days 14_1_ and days 14_2_ was standing for days 14, rat 1 and 2, respectively. Thus, the current data were presented as the mean ± SD of each individual rat with triplicate RTQ-PCR experiments. All the results were analyzed by independent-samples test (t test). Asterisks denote the mean values of RTQ-PCR data that are significantly different between PH groups and SO groups (*; P<0.05 and **; P<0.01). Egf; Epidermal growth factor, LR; Liver regeneration, PH; Partial hepatectomy, SO; Sham-operation and RTQ-PCR; Real time quantitative- polymerase chain reaction.

In order to understand if the change of methylation in the *Egf* promoter region affects the expression of *Egf* gene, real-time quantitative PCR was performed to detect the transcript level of *Egf* gene (Fig .2). For the SO group, *Egf* mRNA level was significantly increased in the re-organization phase (4.03, 3.97, 10.75, 13.00, 5.97, 5.39) compared to the regeneration phase (0.56, 0.30, 0.33, 0.36, 0.37, 0.35, 0.50, 0.22, 0.67) (0 hour expression was assumed as 1, P<0.01). However, for the PH group, there was no significantly change of *Egf* mRNA level in the re-organization phase compared to the regeneration phase; its expression decreased in both phases when compared to normal phase or 0 hour. In addition to methylation, gene expression can be regulated by many factors such as non-code RNA and histone modifications. PH is a serious injury. Usually inflammation is accompanied with tissue injury. EGF has pro-inflammatory function (33). After PH, the decreased *Egf* expression in the regeneration and re-organization phase may help to reduce inflammation level. Comparing to PH, the damage was much limited for SO group. Therefore, the differences of *Egf* mRNA level between SO and PH groups may reflect operationinduced inflammation intensity.

The process of LR has been defined as following phases: initiation phase, proliferation phase, and termination phase, then followed by a slow re-organization phase for several weeks. During the re-organization phase, the newly regenerated liver tissue is gradually remolded and eventually returned to the normal histology of liver tissue (34, 35). The mechanism underlying termination of liver regeneration and the re-organization process is poorly understood. It has been suggested that many signaling factors and pathways are participated in such activity, such as transforming growth factor beta (TGF-β), cytokine and growth factor pathways, and reestablishment of extracellular matrix (36). Our study indicates that methylation of CpGs in the *Egf* promoter region is significantly changed during the LR termination phase to the re-organization phase. It is likely that epigenetic modifications such as methylation change provide signals to guide the progression of LR and re-organization accordingly. It will be helpful to study the mechanism of methylation variation in the *Egf* promoter region for a better understanding of their impact on LR.

**Table 1:** Methylation status at 10 CpG sites within the *Egf* promoter region


CpG position	Percentage of methylation at the indicated time points after rat PH and SO															
	0 hour	2 hours	6 hours	12 hours	24 hours	30 hours	36 hours	72 hours	120 hours	168 hours	Days 14_1_	Days 14_2_	Days 21_1_	Days 21_2_	Days 28_1_	Days 28_2_

		0.0	7.4	5.3	5.6	0.0	10.5	5.0	0.0	5.0	10	20	0	0	10	0
1	7.7	5.0	0.0	20.0	5.3	5.3	10.0	5.0	5.3	5.0	10	0	0	0	30	0
		0.0	3.7	10.5	5.6	0.0	5.3	0.0	0.0	5.0	0	0	0	0	0	10
2	3.8	0.0	5.0	10.0	5.3	5.3	5.0	10.0	0.0	0.0	0	10	0	10	0	0
		56.5	29.6	26.3	11.1	5.0	15.8	5.0	11.1	5.0	60	40	70	60	30	70
3	80.8	60.0	25.0	55.0	52.6	21.1	50.0	35.0	36.8	35.0	50	60	60	60	20	70
		65.2	48.1	78.9	100.0	90.0	89.5	100.0	83.3	95.0	70	60	80	80	50	50
4	73.1	70.0	75.0	60.0	52.6	68.4	40.0	45.0	42.1	45.0	80	70	80	40	70	60
		82.6	70.4	100.0	100.0	100.0	94.7	100.0	100.0	100.0	90	80	80	100	90	80
5	88.5	85.0	90.0	90.0	84.2	84.2	70.0	85.0	89.5	100.0	90	100	90	70	80	80
		69.6	63.0	94.7	100.0	95.0	89.5	100.0	88.9	100.0	100	100	80	100	60	80
6	80.8	90.0	85.0	90.0	84.2	73.7	55.0	70.0	73.7	80.0	90	80	90	60	90	90
		47.8	66.7	84.2	94.4	90.0	78.9	90.0	88.9	90.0	70	90	80	100	40	60
7	69.2	55.0	85.0	85.0	63.2	78.9	65.0	60.0	52.6	60.0	60	70	70	70	50	70
		47.8	74.1	78.9	100.0	90.0	89.5	100.0	88.9	95.0	80	90	90	80	50	60
8	73.1	55.0	85.0	75.0	63.2	89.5	80.0	55.0	68.4	50.0	60	80	80	70	60	90
		30.4	44.4	84.2	83.3	95.0	100.0	95.0	94.4	95.0	40	50	40	80	30	50
9	46.2	55.0	85.0	55.0	68.4	94.7	50.0	55.0	52.6	35.0	60	50	50	70	30	70
		56.5	70.4	84.2	94.4	90.0	89.5	90.0	94.4	95.0	80	70	90	90	50	70
10	57.7	55.0	85.0	75.0	63.2	94.7	85.0	65.0	73.7	50.0	90	70	60	80	60	70


For each CpG site, the upper-row data was generated from PH group and the lower-row from SO group. Data in blue was previously published (28). Right panel provides the results of the current study with days as time points. Each data entry was obtained from one rat, for example, days 14_1_ and days 14_2_ stand for days 14, rat 1 and 2, respectively. Methylation modification of CpGs in the *Egf* promoter region could be restored to the status before PH operation. *Egf*; Epidermal growth factor, PH; Partial hepatectomy and SO; Sham-operation.
